# Shikonin Increases Glucose Uptake in Skeletal Muscle Cells and Improves Plasma Glucose Levels in Diabetic Goto-Kakizaki Rats

**DOI:** 10.1371/journal.pone.0022510

**Published:** 2011-07-26

**Authors:** Anette I. Öberg, Kamal Yassin, Robert I. Csikasz, Nodi Dehvari, Irina G. Shabalina, Dana S. Hutchinson, Mona Wilcke, Claes-Göran Östenson, Tore Bengtsson

**Affiliations:** 1 Department of Physiology, Arrhenius Laboratories F3, The Wenner-Gren Institute, Stockholm University, Stockholm, Sweden; 2 Department of Molecular Medicine and Surgery, Karolinska Institutet, Stockholm, Sweden; 3 Department of Pharmacology, Monash University, Parkville, Victoria, Australia; 4 Monash Institute of Pharmaceutical Sciences, Monash University, Parkville, Victoria, Australia; 5 Glucox Biotech AB, Stockholm, Sweden; New Mexico State University, United States of America

## Abstract

**Background:**

There is considerable interest in identifying compounds that can improve glucose homeostasis. Skeletal muscle, due to its large mass, is the principal organ for glucose disposal in the body and we have investigated here if shikonin, a naphthoquinone derived from the Chinese plant *Lithospermum erythrorhizon,* increases glucose uptake in skeletal muscle cells.

**Methodology/Principal Findings:**

Shikonin increases glucose uptake in L6 skeletal muscle myotubes, but does not phosphorylate Akt, indicating that in skeletal muscle cells its effect is medaited via a pathway distinct from that used for insulin-stimulated uptake. Furthermore we find no evidence for the involvement of AMP-activated protein kinase in shikonin induced glucose uptake. Shikonin increases the intracellular levels of calcium in these cells and this increase is necessary for shikonin-mediated glucose uptake. Furthermore, we found that shikonin stimulated the translocation of GLUT4 from intracellular vesicles to the cell surface in L6 myoblasts. The beneficial effect of shikonin on glucose uptake was investigated *in vivo* by measuring plasma glucose levels and insulin sensitivity in spontaneously diabetic Goto-Kakizaki rats. Treatment with shikonin (10 mg/kg intraperitoneally) once daily for 4 days significantly decreased plasma glucose levels. In an insulin sensitivity test (s.c. injection of 0.5 U/kg insulin), plasma glucose levels were significantly lower in the shikonin-treated rats. In conclusion, shikonin increases glucose uptake in muscle cells via an insulin-independent pathway dependent on calcium.

**Conclusions/Significance:**

Shikonin increases glucose uptake in skeletal muscle cells via an insulin-independent pathway dependent on calcium. The beneficial effects of shikonin on glucose metabolism, both in vitro and in vivo, show that the compound possesses properties that make it of considerable interest for developing novel treatment of type 2 diabetes.

## Introduction

Due to the increasing number of people with type 2 diabetes mellitus (an estimated 285 million people, corresponding to 6.4% of the world's adult population in 2010), there is considerable interest in identifying compounds that can improve glucose tolerance. Extracts from the dried gromwell root (*Lithospermum erythrorhizon*) have been used in traditional Chinese medicine to treat a variety of disorders involving inflammation and cancer and the key active substance in the gromwell root has been shown to be the napthoquinone shikonin [1]. Interestingly, shikonin has been reported to increase glucose uptake in adipocyte 3T3-L1 cells, primary rat adipocytes and cardiomyocytes [2], by enhancing both insulin signaling as well as increasing glucose uptake by itself [2], and inhibiting fat accumulation in 3T3-L1 adipocytes [3]. The mechanisms whereby shikonin increases glucose uptake into adipocytes are not fully understood. Insulin increases glucose uptake in adipocytes and skeletal muscle cells by increasing the translocation of intracellular vesicles containing GLUT4 to the cell surface by a mechanism dependent upon activation of phosphatidylinositol 3-kinase (PI3K) and Akt [4]. Previous studies have shown that shikonin increases glucose uptake in adipocytes independently of the insulin receptor, IRS proteins and PI3K but through an unidentified tyrosine kinase mediated mechanism requiring Akt phosphorylation [2]. In CHO cells transfected with the insulin receptor, shikonin inhibited PTEN which may explain some of its insulin like actions such as increased glucose uptake [5].

Glucose uptake into muscle fibers provides the cells with important energy substrate and has major impact on whole body glucose homeostasis. Thus there are several signals, extra- as well as intracellular, that can regulate glucose uptake in muscle cells.

The major hormone regulating glucose uptake in skeletal muscles is insulin that is secreted after a meal in order to decrease blood glucose levels. When insulin binds the insulin receptor it induces an intracellular signaling cascade, resulting in translocation of the glucose transporter GLUT4 to the plasma membrane and consequently increased glucose uptake. However, in type 2 diabetes, insulin sensitivity is decreased in extrahepatic tissues, mainly skeletal muscle, leading to markedly impaired insulin activation of the insulin signaling cascade and GLUT4-translocation. Thus it is of great importance to find and characterize insulin-independent pathways stimulating glucose uptake in skeletal muscle. For example, the hormone epinephrine and the neurotransmitter norepinephrine can induce glucose uptake in skeletal muscles independently of insulin. These molecules can act both on α- and β-adrenoceptors and further characterization of the signaling shows that both receptor subtypes, although utilizing different intracellular signaling cascades, will mediate glucose uptake through Akt independent pathways [6], [7]. Furthermore, activation of the M3 muscarinic acetylcholine receptor can induce glucose uptake in myotubes by a CaMKK-AMPK-dependent mechanism [8].

Other intracellular signalling intermediates besides PI3K/Akt can affect glucose uptake e.g. AMPK (AMP activated protein kinase), ROS and hypoxia. The most well described of these signals is AMPK, AMP activated kinase, which functions as an energy sensor. It can be activated by several different mechanisms, including an increased AMP/ATP-ratio, allosteric activation by AMP or or via activation of 3 identified upstream kinases (AMPK-kinase) [9]. AMPK can regulate glucose uptake due to stress, via activation of GPCRs (see [10]) or after activation by pharmacological agents such as 5-aminoimidazole-4-carboxamide 1 β-D-ribonucleoside (AICAR) which is used widely as a pharmacological activator of AMPK. Importantly increases in glucose uptake through AMPK activation are sought for the treatment of type 2 diabetes since the AMPK signaling pathway is not down regulated in type 2 diabetes (as opposed to the insulin signaling pathway) and pharmacological agents such as metformin used clinically for the treatment of type 2 diabetes exert some of their actions through AMPK.

Contraction of skeletal muscles (such as that occurring during exercise) also leads to increased glucose uptake. This is in part due to AMPK-activation but also on non-AMPK mediated mechanisms which may include ROS and NO production [11], and importantly increases in intracellular Ca^2+^ levels[12]. Furthermore, increased calcium levels can induce glucose uptake also independently of contraction [13] and calcium ionophores are shown to increase glucose uptake in in L6 skeletal muscle cells as well as in primary skeletal muscle myoblast cultures [14], [15].

There are several mechanisms whereby Ca^2+^ can induce glucose uptake. Ca^2+^ and calcium-calmodulin dependent protein kinases (CaMKKs) can, be upstream regulators of both AMPK- and Akt-activity, but there is evidence that calcium can increase glucose uptake independently of AMPK or Akt. For example, Ca^2+^-elevating agents such as caffeine increase glucose uptake in skeletal muscle by a mechanism additive to AMPK-activation [16], inhibition of CaMKII via *in vivo* electroporation of CaMKII inhibitory peptide into mouse tibialis anterior muscles decreased contraction-induced muscle glucose uptake significantly without affecting phosphorylation of Akt-substrates or AMPK [17] and expression of constitutively active CaMKKα in mouse muscles increased glucose uptake 2.5 fold without affecting Akt-phosphorylation. This effect on glucose uptake was also seen in mice with imparied AMPK-function, indicating that there is an AMPK-independent pathway [18]. Thus calcium can induce glucose uptake in muscles via both AMPK-dependent and –independent mechanisms.

We examined the possible effect of shikonin on glucose uptake in skeletal muscle as well as on total body glucose homeostasis. Skeletal muscle, due to its large mass, is the principal organ for glucose disposal in the body and therefore effects on skeletal muscle cells can have profound effects on glucose homeostasis. We have utilized the L6 skeletal muscle cell line (commonly used for studies of glucose uptake [19]) to investigate whether shikonin affects glucose uptake and the mechanism whereby this occurs. Further, we have investigated the effect of shikonin on plasma glucose levels and insulin tolerance in diabetic animals, using the Goto-Kakizaki (GK) rat as a model of non-obese type 2 diabetes [20], [21]. The GK rat develops hyperglycemia post-natally and maintains moderately enhanced plasma glucose levels during its lifetime. Like in human type 2 diabetes, the glucose intolerance in the GK rat is due partly to impaired insulin secretion, but also to reduced insulin sensitivity in target tissues. We show that shikonin increases GLUT4-translocation and glucose uptake in L6 skeletal muscle cells, independently of Akt-AMPK but dependent upon calcium increases, and improves glucose tolerance *in vivo*.

## Materials and Methods

### Animals

Diabetic Goto-Kakizaki (GK) male rats were bred at the Department of Molecular Medicine and Surgery (Karolinska Institute, Stockholm, Sweden) [20]. The animals were kept at 22°C with free access to food and water. All experiments were conducted with the permission of the North Stockholm Animal Ethics Committee. Six male GK rats, 6 weeks old and weighing 100–200 g, were initially given intraperitoneally (i.p.) vehicle (DMSO/olive oil) (9∶1), once daily for 4 days. In the same 6 rats after one week of the vehicle experiments, shikonin (20 mg/ml) at a dose of 10 mg/kg (500 µl/kg) was injected i.p. once daily at 9 am for 4 subsequent days. Rats were unanaestesized during the whole procedure. Plasma samples were obtained by tail snipping. Plasma glucose levels were measured by glucometer (Accu- Chek Aviva). On the 4^th^ day (one hour after the i.p. injection of the vehicle and shikonin), insulin (Actrapid) was injected subcutaneously (s.c.) at a dose of 0.5 U/kg, and plasma glucose levels were measured before the injection of insulin and every 15 min for 2 hours and then every 30 minutes for 2 hours.

### Ethics Statement

Approved by the Stockholm North Ethical Committee on Animal Research (Dnr N65/06).

### Cell culture

L6 cells (from ATCC) were grown in DMEM (4.5 g/liter glucose) supplemented with 10% (vol/vol) fetal bovine serum, 10 mM HEPES buffer, 2 mM L-Glutamine, 50 U/ml penicillin and 50 µg/ml streptomycin at 37°C with 8% CO_2_. Upon reaching confluence, differentiation was induced by media containing 2% (v/v) fetal bovine serum for 7 days.

### 2-Deoxy-[^3^H]-D-glucose uptake assay

Glucose uptake in L6 cells was measured using the 2-deoxy-[^3^H]-D-glucose method [22] with some modifications [14], except that for cells treated for 20 h, cells were treated with the appropriate drug in serum free media for 19 h 30 min, cells washed in warm phosphate-buffered saline, and media replaced to glucose free media for 10 min before adding 50 nM 2-deoxy-[^3^H]-D-glucose for 6–10 min. When inhibitors were used, the time indicated with the results represents the time cells were pre-equilibrated with the inhibitors before agonists were added.

### Western blot analysis for AMPK and Akt

Cells were serum-starved overnight before each experiment and exposed to drugs for times and concentrations indicated with the data. Cells were harvested and run on polyacrylamide gels as described previously [23] and electro transferred to Hybond-P PVDF membranes (pore size 0.45 µm; Amersham Pharmacia Biotech). Primary antibodies used were AMPK, phospho-AMPK (Thr172), Akt, phospho-Akt (Ser473), phospho-Akt (Thr308), (diluted 1∶1000 except phospho-AMPK diluted 1∶500), which were detected using a secondary antibody (HRP linked anti-rabbit IgG, diluted 1∶2000) and chemiluminescence (ECL, Amersham Pharmacia Biotech). All antibodies were purchased from Cell Signalling.

Quantification of blots was preformed by the software MacBiophotonics ImageJ (McMaster Biophotonics Facility, McMaster University, Canada).

### ATP level measurements

L6-myotubes were serum starved over night, new medium was added for 2 h before stimulation with drugs for 2 h. Cell extracts were isolated and the AMP-to-ATP ratio was measured as previously described [24]. Results are expressed as nanomoles ATP per milligram protein.

### Oxygen consumption

Differentiated L6 cells were treated for 2 h with 1 µM shikonin and harvested by trypsin digestion. Oxygen consumption rates were monitored with a Clark-type oxygen electrode (Yellow Springs Instruments Company, Yellow Springs OH, U.S.A.) in a sealed chamber at 37°C. L6 cells were added to a magnetically stirred oxygen electrode chamber containing DMEM in a final volume of 1.1 ml. The output signal from the oxygen electrode amplifier was collected by a PowerLab/ADInstrument (application program Chart v5.1.1.). The Chart data files were converted to absolute values, based on an oxygen content of 217 nmol of O_2_ per 1 ml of water, and on the number of cell used. For calculation of stable oxygen consumption rates, mean values during about 1 min were obtained from these recordings.

Mitochondria from mouse skeletal muscles were prepared and analysed as previously described [25].

### Ca ^2+^-measurements

Cytosolic free [Ca^2+^] ([Ca^2+^]_i_) was measured during exposure to shikonin (1 µM) or insulin (1 µM) using the fluorescent ratiometric Ca^2+^ indicator indo-1. Cells were loaded with indo-1 by exposing them to indo-1-AM (8.3 µM, Invitrogen) for 20 minutes followed by at least 20 minutes of washing. Indo-1 was excited with light at 360 nm, and light emission at 405±5 and 495±5 nm was measured with two photomultiplier tubes. The ratio (R) of the light emission at 405 nm to that at 495 nm was converted to [Ca^2+^]_i_ as described previously [26]. After establishing a baseline, shikonin or insulin was added.

### Immunocytochemistry

L6 cells stably transfected with GLUT4myc (kindly provided by Prof. Amira Klip, University of Toronto, Canada) were plated in 4 well culture chamber slides (BD Biosciences, Franklin Lakes, BJ). Cells were serum-starved 16 h before stimulation with insulin or shikonin. Cells were washed with PBS after stimulation and fixed for 5 min with 4% formaldehyde in PBS, and quenched with 50 mM glycine in PBS for 10 min. Cells were blocked with 5% BSA in PBS for 1 h and incubated with Myc-tag primary antibody solution (1∶200 dilution in 1.5% BSA in PBS) at 4°C overnight followed by washing with PBS and 1 h incubation at room temperature with Alexa Fluor 488-conjugated goat anti-rabbit IgG secondary antibody (1∶500 dilution, 1.5% BSA in PBS). Slides were washed with PBS and mounted with ProLong Gold antifade reagent (Invitrogen). Images were observed in a Confocal microscope, ZEISS LSM 510 META. Quantification of immunocytochemistry image was performed by using ImageJ program (NIH).

### Data analysis

All experiments were performed in singlicate or duplicate with *n* referring to the number of independent experiments performed. All results are expressed as mean ± S.E.M. of *n.* Statistical analysis was made with one-way ANOVA, using Tukey post hoc-test or paired Student's t-test, using Bonferroni's correction for multiple testing when appropriate.

### Drugs and reagents

Shikonin was synthesised by Interchem (NJ, USA). Drugs and reagents were purchased as follows: 2-deoxy-[^3^H]-D-glucose (12Ci/mmol, Amersham Biosciences, Buckinghamshire, UK); insulin (Actrapid®) (Novo Nordisk, Bagsvaerd, Denmark); FCCP (carbonyl cyanide p-trifluoromethoxyphenylhydrazone), 0.25% trypsin in HBSS (Sigma); Fatty acid-free bovine serum albumin, fraction V (Roche Diagnostics GmbH, Germany). All cell culture medium and supplements were obtained from Sigma. All antibodies were obtained from Cell Signaling Technology (Beverly, MA, U.S.A.).

## Results

### Shikonin increases glucose uptake L6 myotubes

**Figure 1:**
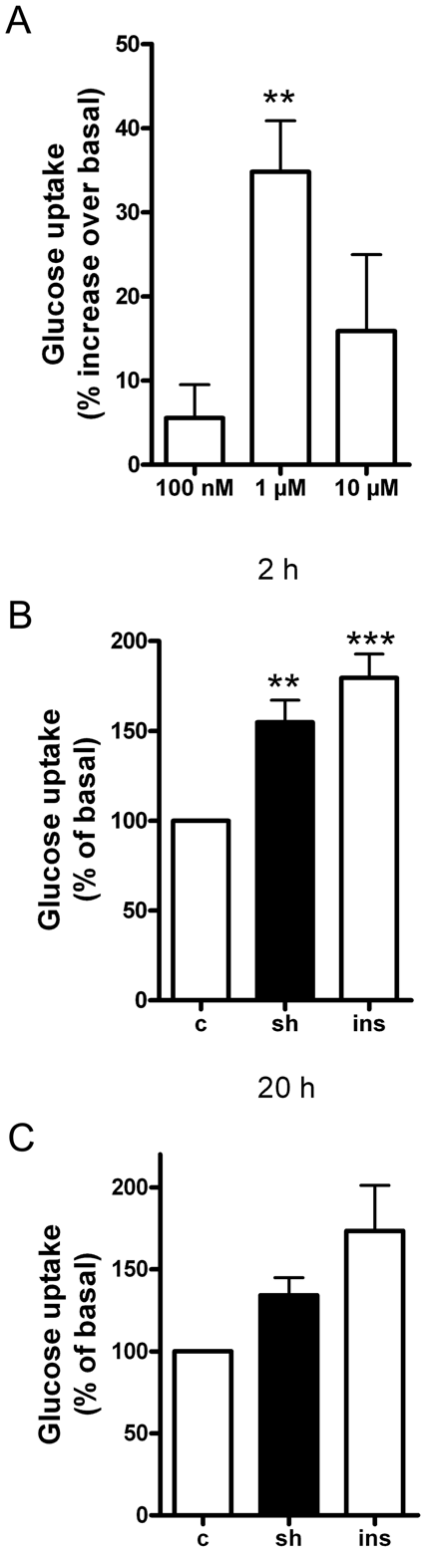
Shikonin increases glucose uptake in L6 myotubes. (**A**) Glucose uptake in differentiated L6 myotubes in response to 100 nM, 1 µM or 10 µM shikonin treatment (2 h). In (**B**) and (**C**), cells were treated with 1 µM shikonin (sh) or 1 µM insulin (ins) for 2 h or 20 h respectively). Graphs show mean ± SEM of four (A), seven (B) or three (C) experiments. Asterisks represent statistical difference as analyzed by one way ANOVA between basal and treated cells (** P<0.01 *** P<0.001).

Shikonin increased glucose uptake (following 2 h of treatment) in a concentration-dependent manner (Fig. 1A). A concentration of 1 µM was found to give the highest increase (p<0.01) while higher concentrations of shikonin (≥10 µM) were found to cause cell detachment (data not shown). This concentration of shikonin (1 µM) is in the same order of magnitude as found to give the highest glucose uptake response in rat cardiomyocytes, but lower than that found for 3T3-L1 adipocytes [2], indicating muscles to be highly sensitive to shikonin. Stimulation with shikonin (1 µM) increased glucose uptake in L6-myotubes following 2 h of shikonin treatment (p<0.01; Fig. 1B), which was the same magnitude of response as that obtained following insulin treatment (1 µM, p<0.01; Fig. 1B). Longer term treatment of cells with either shikonin (1 µM) or insulin (1 µM) for 20 h showed no significant increase in glucose uptake at this time point (there was a tendency of shikonin and insulin to increase glucose uptake but this was not significantly different from control, indicating that the shikonin effect is within hours and not long-term (Fig. 1C)).

### Effect of shikonin on the phosphorylation of Akt

**Figure 2:**
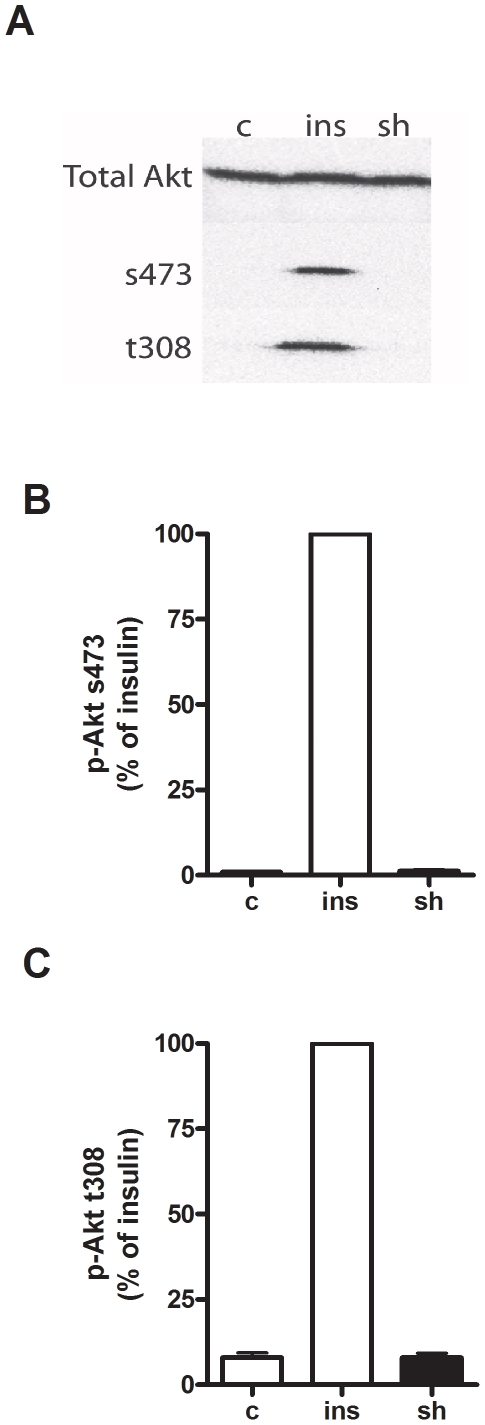
Akt-phosphorylation in response to shikonin and insulin. (**A**) Immunoblot of total Akt or phosphorylated Akt at serine 473 (s473) or threonine 308 (t308) following treatment of L6 myotubes with water as control (c) or stimulated with either 1 µM shikonin (sh) or 1 µM insulin (ins) for 2 h. Figure is representative of three independent experiments performed. (**B**) and (**C**) show quantification of immunoblots expressed as a percentage of the response to 1 µM insulin (ins) for 2 h.

Shikonin increases Akt-phosphorylation in 3T3-L1 cells [2], thus we wanted to investigate if shikonin also affected Akt phosphorylation in L6 cells. In L6 myotubes shikonin treatment (1 µM, 2 h) did not affect phosphorylation of Akt at residues s473 or t308 , as opposed to insulin (1 µM, 2 h) treatment which increased phosphorylation of Akt at both residues (Fig. 2). This indicates that in L6 myotubes, shikonin increases glucose uptake independent of Akt-phosphorylation and thus act via a pathway different than that of insulin.

### Effect of shikonin on the phosphorylation of AMPK

**Figure 3:**
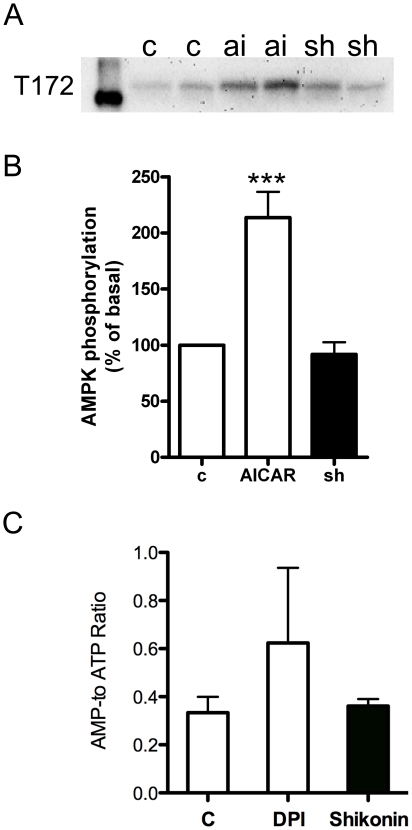
Shikonin does not affect AMP-phosphorylation or ATP/ADP-ratio. (**A**) Immunoblot phosphorylated AMPK at threonine 172 (t172) following treatment of L6 myotubes with water as control (c) or stimulated with either 1 µM shikonin (sh) or 2 mM AICAR (ai) for 2 h. Figure is representative of six independent experiments performed.(**B**) Quantification of AMPK phosphorylation (t172) expressed as a percentage of the basal levels. Asterisks represent statistical difference as analyzed by one way ANOVAbetween basal and treated cells (*** P<0.001). Graph show mean ± SEM (n = 6). (**C**) AMP to ATP-ratio in L6 myotubes 1 µM shikonin or 1 µM DPI treatment. Graph show mean ± SEM (n = 4).

One of the major insulin-independent regulators of glucose uptake is AMPK. Thus we wanted to investigate the possible activation of AMPK by shikonin. In L6 myotubes shikonin treatment (1 µM, 2 h) did not affect phosphorylation of AMPK at residue t172 as opposed to the pharmacological activator of AMPK, AICAR (2 mM, 2 h), which increased AMPK phosphorylation by ∼2 fold (Fig. 3A and 3B).. Conversely, shikonin (1 µM for 2 h) had no effect ont the AMP/ATP-ratio which was increased by diphenyleneiodonium (DPI, 1 µM), which has previously been shown to increase AMPK phosphorylation in L6 cells through increases in the AMP/ATP ration [27]((Fig. 3C).

### Effect of shikonin on oxygen consumption in L6 myotubes

**Figure 4:**
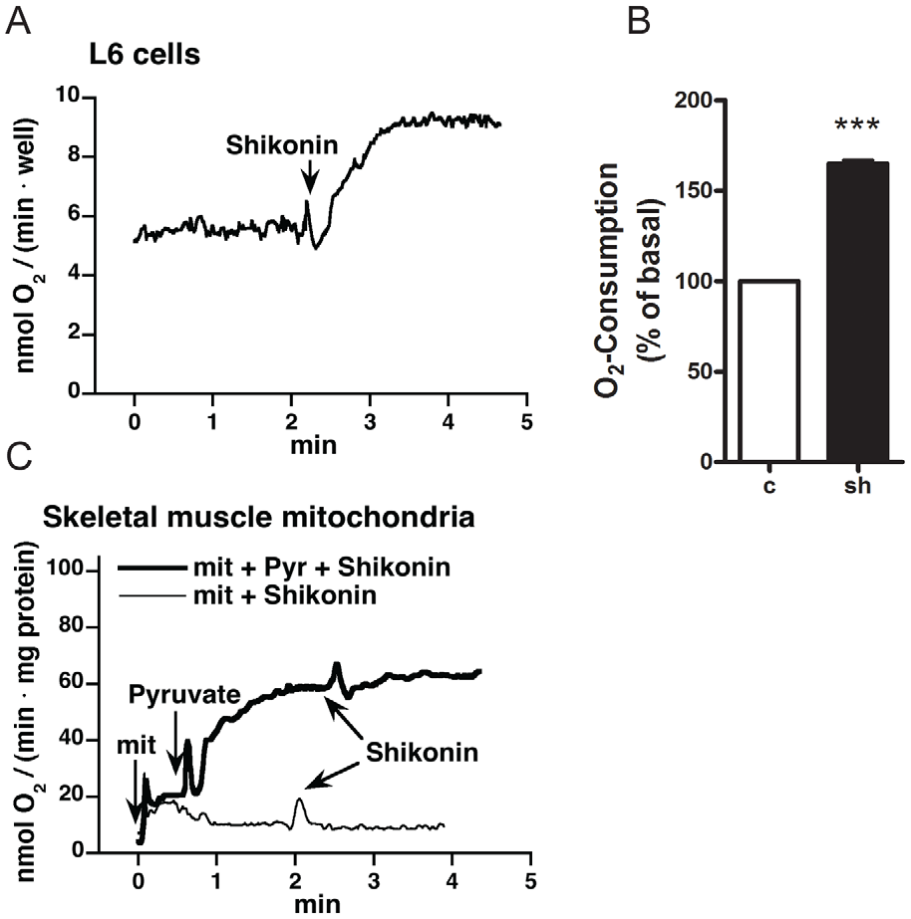
Effects of shikonin on oxygen consumption. (**A**) Oxygen consumption measurements in L6 myotubes. Representative graph from three independent measurements of oxygen consumption in L6 myotubes.1 µM shikonin was added as indicated. (**B**) Shikonin stimulated increase in oxygen consumption in L6 myotubes expressed as percentage of basal. Graphs show mean ± SEM from three independent experiments. (**C**) Oxygen consumption in mitochondria from skeletal muscle in response to shikonin was measured in the absence (thin line) or presence (bold line) of pyruvate. 10 µM shikonin was added as indicated.

To investigate the effect of shikonin on overall cell metabolism and the involvement of mitochondria, oxygen consumption rates were monitored with a Clark-type oxygen electrode. Addition of 1 µM shikonin increased oxygen consumption in intact L6 muscle cells (Fig. 4A). This acute effect was seen also when adding 1 µM shikonin to cells that had been incubated with shikonin for 2 h (in these experiment the final concentration of shikonin was 2 µM). The average increase from three independent experiments was 165±2% over basal (Fig. 4B). To test if this was due to uncoupling and direct effect on mitochondria, measurement was performed on mitochondria isolated from mouse skeletal muscle. Addition of shikonin did not alter the rate of oxygen consumption in mitochondria, either in the presence or the absence of the substrate pyruvate indicating that shikonin does not act directly on mitochondria (Fig. 4C).

### Dependence of calcium on shikonin-mediated glucose uptake

**Figure 5:**
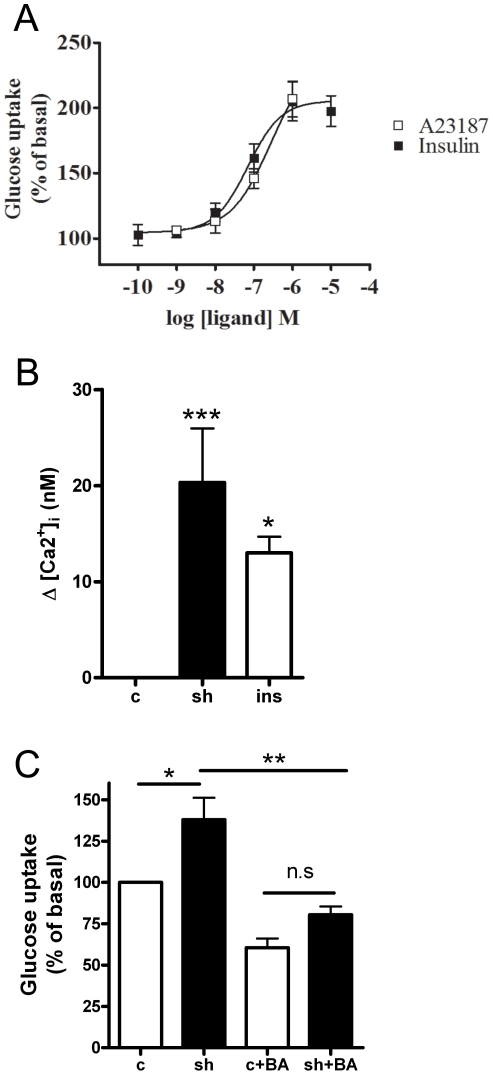
Calcium is involved in shikonin-medaited glucose uptake. (**A**) Insulin and A23187 stimulated glucose uptake in L6 myotubes. Data are mean ± SEM of three experiments (**B**) Measurement of intracellular calcium levels by the fluorescent ratiometric Ca^2+^ indicator indo-1 following acute treatment with shikonin (1 µM) or insulin (1 µM). ***p<0.001, *p<0.05, one way ANOVA . Graph show mean delta value ± SEM of 15 experiments performed. Asterisks represent statistical difference as analyzed by one way ANOVA between basal and treated cells (*p<0.05, *** P<0.001). (**C**) Effect of BAPTA-AM on glucose uptake mediated by shikonin in L6 mytoubes. Cell were stimulated with shikonin for 2 h in the absence or presence of 5 µM 1,2-Bis(2-aminophenoxy)ethane-N,N,N′,N′-tetraacetic acid tetrakis(acetoxymethyl ester), BAPTA (BA). Graph show mean ± SEM of 3 experiments performed. Asterisks represent statistical difference as analyzed by one way ANOVA (*p<0.05, **<0.01).

One possible mechanism to cause a rapid activation of oxygen consumption is by altering the intracellular concentrations of Ca^2+^ [28]. Increasing levels of A23187 (a calcium ionophore) increased glucose uptake in a concentration-dependent manner in L6 muscle cells to the same levels as insulin (Fig. 5A; concentrations higher than 1 µM of A23187 resulted in cell detachment (data not shown)). To examine if shikonin increased calcium levels in L6 myotubes we utilized a fluorescent ratiometric Ca^2+^-indicator, indo-1. Shikonin gave a significant increase (p<0.001) in intracellular calcium levels within minutes of shikonin addition to cells (Fig. 5B and Table 1), as did insulin (1 µM, p<0.05). The changes in calcium levels caused by shikonin and insulin were somewhat different; while shikonin increased calcium almost instantly and to a higher degree; the effect of insulin was more delayed (not shown).

**Table 1:** Shikonin increases free calcium in L6-myotubes.

	Resting (nM)	Peak (nM)	Delta (nM)	P-value
**Insulin**	92±9	105±10	13±2	<0.001
**Shikonin**	78±7	98±9	20±6	<0.005

Intracellular levels of calcium in L6 cells before and after acute exposure to 1 µM insulin or shikonin. Data are means ± SEM (n = 15).

To investigate the role of calcium in shikonin–mediated glucose uptake, glucose uptake was assessed in the absence and presence of the non-cell permeable calcium chelator 1,2-bis(2-aminophenoxy)ethane-N,N,N′,N′-tetraacetic acid tetrakis acid (BAPTA) and its cell-permeable analogue 1,2-Bis(2-aminophenoxy) ethane-N,N,N′,N′-tetraacetic acid tetrakis-acetoxymethyl ester (BAPTA-AM). When reducing extracellular calcium with BAPTA (5 µM, 5 min before stimulation), the effect of shikonin on glucose uptake was unaltered (data not shown). In contrast, the cell permeable analogue BAPTA-AM, (5 µM, 5 min before stimulation) reduced shikonin stimulated glucose uptake in L6 myotubes (p<0.05) (Fig. 5C).

### Detection of GLUT4-translocation

**Figure 6:**
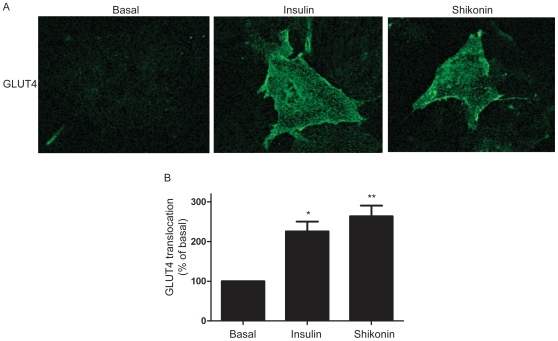
Shikonin stimulates translocation of GLUT4. (**A**) Representative confocal image of GLUT4-translocation which was detected by myc-antibody in cells stable transfected with GLUT4myc after 2 h treatment with either 1 µM shikonin or 1 µM insulin (**B**) Quantification of confocal images obtained in (A) by using Image J and expressed as % of basal. Graph show mean ± SEM of 3 experiments performed. Asterisks represent statistical difference as analyzed by one way ANOVA between basal and treated cells (***p<0.001, *p<0.05).

There are several possible molecular mechanisms which lead to increased glucose uptake into cells, of which the most well-characterized in skeletal muscle is translocation of glucose transporter GLUT4. In order to test if shikonin could affect GLUT4-translocation, immunocytochemistry was performed in L6 myoblasts. Insulin significantly stimulated GLUT4-translocation compared to basal (∼2.5 fold increase in cell surface GLUT4 p<0.05). Shikonin in itself also significantly induced GLUT4 translocation (p<0.01) to the same extent as insulin (Fig. 6).

### Shikonin administration to GK rats

**Figure 7:**
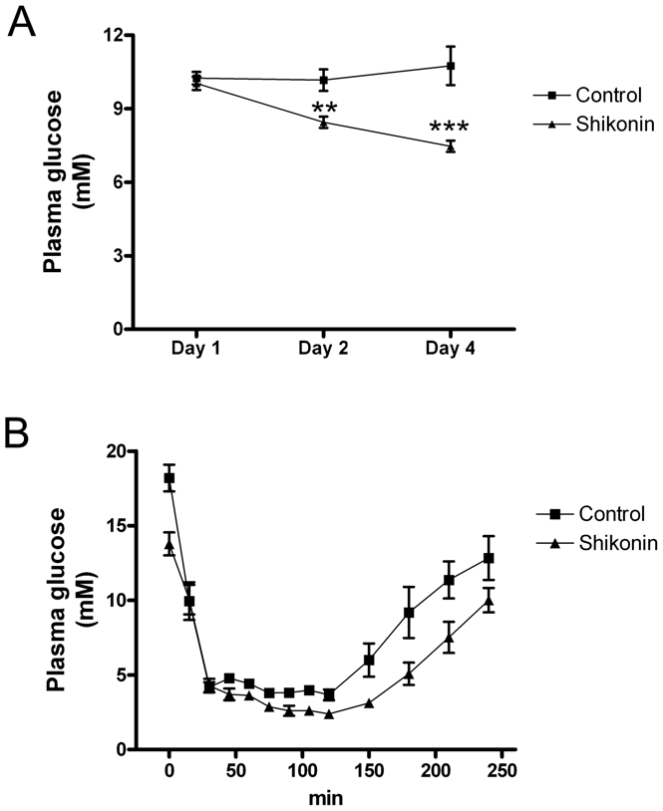
Shikonin injections lower plasma glucose levels in diabetic GK-rats. (**A**) Shikonin effect on plasma glucose in GK-rats treated with DMSO/oil (squares) or shikonin (10 mg/kg intraperitoneally) (triangles) for 4 days. Results are mean ±SEM of 6 rats.**p< 0.01 for shikonin day 2 compared to day 1, ***p<0.001 for shikonin day 4 compared to day 1 (Students t-test). (**B**) Shikonin effect on insulin sensitivity in GK-rats. Plasma glucose was measured in GK-rats after s.c. insulin in GK rats treated with DMSO/oil (squares) or shikonin (triangles) for 4 days. Results are mean ±SEM of 6 rats.

To test the effect of shikonin on glucose homeostasis, GK rats were used. During four days, GK-rats were treated with a daily i.p. injection of shikonin (10 mg/kg). In the rats treated with shikonin and placebo, the general condition, e.g. alertness and physical activity, was observed to be normal during the whole experiment. Plasma glucose levels were significantly lowered on 2^nd^ and 4^th^ days compared to day 1(Fig. 7A). On the first day before i.p. shikonin injection, the plasma glucose was 10.0±0.3 mM, and the plasma glucose levels of the other consecutive days were 8.5±0.2and 7.5±0.2 mM, respectively (p<0.01 and p<0.001 compared to the first day). Also the areas under the glucose curve (AUC) were significantly lower in shikonin treated rats compared to control rats (−4.9±0.8 vs 0.4±1.0-mM/3d, p = 0.014).

In an insulin tolerance test, administration of insulin reduced plasma glucose concentrations in all rats. The plasma glucose levels prior to insulin injection were lower in the shikonin treated rats compared to the control (p = 0.007; Fig. 7B). Between 30–240 min after injection of insulin, the AUCs were 39.3±105.9 and 536.4 ±144.0 mM/210 min, respectively (p<0.02).

## Discussion

In this study we have investigated the effect of shikonin on glucose uptake in L6 myotubes and examined its *in vivo* effect on plasma glucose levels in diabetic GK-rats. We found that shikonin increased glucose uptake to the same extent as insulin in L6 myotubes. In contrast to previous studies showing that shikonin increases Akt-phosphorylation in 3T3-L1 adipocytes and CHO-cells transfected with the insulin receptor [2], [5], we found that in L6 myotubes shikonin did not affect Akt-phosphorylation. These results suggest that shikonin increases glucose uptake in myotubes through a pathway different from that of insulin. One major insulin-independent pathway regulating glucose uptake is AMPK. AMPK functions as an energy sensor that can increase glucose uptake and fatty acid oxidation when energy levels in a cell are low. Due to its effect on glucose uptake, AMPK is a novel site for the treatment of type 2 diabetes. However, in the present study, measurement of AMP/ATP-levels showed no difference between control and shikonin treated myotubes, nor was any increase in phosphorylation of AMPK detected following shikonin treatment observed, indicating that shikonin does not act via AMPK.

To examine whether shikonin increased glucose uptake in L6 cells through an increase in cellular respiration and thus an elevated need for substrate, we tested if oxygen consumption rates from L6 cells or isolated mouse skeletal muscle mitochondria were altered following shikonin treatment. When adding shikonin to L6 cells, the rate of oxygen consumption rapidly increased. This is likely to lead to increased glucose uptake and could either be due to increased cellular respiration or an uncoupling effect on the mitochondria. Since shikonin had no effect on isolated mousce skeletal muscle mitochondria, it is not likely to be an uncoupler itself. One way to change mitochondria function is to change ion-gradients, and accumulation of Ca^2+^ into mitochondria has been shown to cause a transient depolarisation of the mitochondria membrane potential [28]. Measurement of intracellular free calcium in L6 myotubes showed that shikonin treatment in fact leads to an increase in free calcium levels. This is very interesting since increased calcium levels are considered to be one of the major events in contraction-mediated glucose uptake in skeletal muscle [12]. Furthermore, calcium is shown to increase glucose uptake in muscle cells also independently of contraction [13]. We show that the calcium ionophore A23187 increases glucose uptake in L6 cells, confirming previous results from both cell lines and primary myoblast cultures [14], [15]. In our study we also found that insulin treatment increased intracellular calcium levels. The role of calcium in insulin signaling is debated, but there are several studies showing calcium to be important for late steps in the insulin signaling cascade, enabling docking and fusion of GLUT4-containing vesicles to the plasma membrane [30]. Studies in single muscle fibers from mice show that insulin can increase calcium levels close to the plasma membrane without affecting the global levels of free calcium [31]. This means that even a small increase in whole cell calcium levels possibly reflects a substantially local increase. In order to investigate if the calcium-effect caused by shikonin leads to increased glucose uptake, cells were pretreated with the calcium chelator BAPTA and its cell permeable analogue BAPTA-AM before stimulation with shikonin. Treatment with BAPTA-AM (but not BAPTA) abolished the shikonin mediated glucose uptake, suggesting shikonin to cause glucose uptake by increasing intracellular calcium levels.

There are several possible molecular mechanisms whereby to increase glucose uptake into cells. The most well-studied of those is the translocation of the glucose transporter GLUT4. Upon insulin-stimulation, as well as after AMPK-activation or muscle contraction, GLUT4 is translocated from intracellular storages to the plasma membrane. Surprisingly, we found that shikonin treatment induced GLUT4-translocation in muscle cells to the same extent as caused by insulin. This is a very exciting finding, providing molecular proof of the end-mechanism utilized by shikonin to increase glucose uptake. GLUT4-translocation has previously been suggested to be calcium dependent, both in insulin-action, as discussed above, as well as GLUT4-translocation due to muscle contraction [29]. We suggest here that the increased calcium levels after shikonin treatment is important for GLUT4-translocation although this is not investigated in this study. We believe the effect of shikonin on GLUT4 translocation is an important finding, because in the light of the increasing prevalence of type 2 diabetes it is vital to find and characterize insulin-independent pathways leading to glucose uptake. Impaired insulin signaling and GLUT4-translocation in peripheral tissues is a major hallmark in diabetes. Interestingly, our current findings show that shikonin uses the same end mechanism as insulin (GLUT4-translocation), without using the same intracellular signal (Akt-phosphorylation). Thus, there is a possibility that shikonin could affect glucose uptake also in insulin-insensitive muscles.

We formulated the hypothesis that shikonin could have beneficial effect on plasma glucose in diabetic animals and chose to test our hypothesis in diabetic GK rats. GK rats were injected with shikonin daily for 4 days. No changes were observed in general health conditions of the shikonin-treated rats, indicating shikonin not to be detrimental to the animals, although we did not examine possible toxic effects of long-term treatment. After 2 and 4 days of daily injection, lower plasma glucose levels were found in shikonin treated rats. This finding shows for the first time that shikonin has effects on glucose homeostasis. On day 4, insulin injection lowered plasma glucose in both groups. However, the absolute plasma glucose levels were lower in the shikonin treated rats during the whole experiment. Thus, the effects of shikonin and insulin are additive, pointing in a direction that shikonin and insulin acts via different pathways. Thus the current data supports our conclusions from skeletal muscle cells.

Although our main focus has been on skeletal muscle, it is possible that shikonin has several beneficial effects in diabetic animals. Since shikonin has previously been reported to increase glucose uptake and enhance insulin signaling in 3T3-L1 and primary adipocytes [2], and inhibit fat accumulation in 3T3-L1 adipocytes [3], it is possible that in addition to the effects in muscle, shikonin increases insulin sensitization in fat tissue. Furthermore, shikonin is shown to inhibit NADPH-oxidase, a membrane bound enzyme complex that catalyses the formation of ROS in leukocytes [32], and such inhibition could potentially increase the insulin sensitivity due to a suppression of inflammation [33]. However, our present data suggest that the major effect of shikonin *in vivo* did not increase insulin-sensitization. The fold change in plasma glucose levels after insulin injection in GK-rats is in the same order of magnitude for the shikonin-treated animals as for the control. This would indicate that insulin stimulates glucose uptake to the same degree in control and the treated animals. This, together with our results from L6 cells, as well as the fact that skeletal muscle is a major site for glucose clearing, suggests that the main effect of shikonin, to decrease plasma glucose levels *in vivo*, is through glucose uptake in skeletal muscle.

In conclusion, we have shown that shikonin increases glucose uptake in L6 skeletal muscle cells by an insulin-independent mechanism involving increased intracellular calcium levels and GLUT4-translocation. Since insulin signaling is impaired in type 2 diabetes it is of great interest to find molecules and mechanisms that regulates glucose uptake in peripheral tissues. Due to the large proportion of skeletal muscle in the body, inducing glucose uptake in this tissue can have large effects on plasma glucose levels. Our results indeed show that shikonin has beneficial effects on plasma glucose levels in diabetic GK rats, indicating that this compound or its derivates could be important molecules in the search for novel therapy of type 2 diabetes.

## References

[1] Papageorgiou, VP, Assimopoulou AN, Couladouros EA, Hepworth D, Nicolaou KC (1999). The chemistry and biology of alkannin, shikonin, and related naphthazarin natural products.. Angew Chem Int Ed.

[2] Kamei R, Kitagawa Y, Kadokura M, Hattori F, Hazeki O (2002). Shikonin stimulates glucose uptake in 3T3-L1 adipocytes via an insulin-independent tyrosine kinase pathway.. Biochem Biophys Res Commun.

[3] Lee H, Kang R, Yoon Y (2010). Shikonin inhibits fat accumulation in 3T3-L1 adipocytes.. Phytother Res.

[4] Klip A (2009). The many ways to regulate glucose transporter 4.. Appl Physiol Nutr Metab.

[5] Nigorikawa K, Yoshikawa K, Sasaki T, Iida E, Tsukamoto M (2006). A naphthoquinone derivative, shikonin, has insulin-like actions by inhibiting both phosphatase and tensin homolog deleted on chromosome 10 and tyrosine phosphatases.. Mol Pharmacol.

[6] Hutchinson DS, Bengtsson T (2005). alpha1A-adrenoceptors activate glucose uptake in L6 muscle cells through a phospholipase C-, phosphatidylinositol-3 kinase-, and atypical protein kinase C-dependent pathway.. Endocrinology.

[7] Nevzorova J, Evans BA, Bengtsson T, Summers RJ (2006). Multiple signalling pathways involved in beta2-adrenoceptor-mediated glucose uptake in rat skeletal muscle cells.. Br J Pharmacol.

[8] Merlin J, Evans BA, Csikasz RI, Bengtsson T, Summers RJ (2010). The M3-muscarinic acetylcholine receptor stimulates glucose uptake in L6 skeletal muscle cells by a CaMKK-AMPK-dependent mechanism.. Cell Signal.

[9] Hardie DG, Carling D (1997). The AMP-activated protein kinase—fuel gauge of the mammalian cell?. Eur J Biochem.

[10] Hutchinson DS, Summers RJ, Bengtsson T (2008). Regulation of AMP-activated protein kinase activity by G-protein coupled receptors: Potential utility in treatment of diabetes and heart disease.. Pharmacol Ther.

[11] Merry TL, McConell GK (2009). Skeletal muscle glucose uptake during exercise: A focus on reactive oxygen species and nitric oxide signaling.. IUBMB Life.

[12] Rose AJ, Richter EA (2005). Skeletal muscle glucose uptake during exercise: How is it regulated?. Physiology (Bethesda).

[13] Youn JH, Gulve EA, Holloszy JO (1991). Calcium stimulates glucose transport in skeletal muscle by a pathway independent of contraction.. Am J Physiol.

[14] Hutchinson DS, Bengtsson T (2005). alpha1A-adrenoceptors activate glucose uptake in L6 muscle cells through a phospholipase C-, phosphatidylinositol-3 kinase-, and atypical protein kinase C-dependent pathway.. Endocrinology.

[15] Schudt C, Gaertner U, Pette D (1976). Insulin action on glucose transport and calcium fluxes in developing muscle cells in vitro.. Eur J Biochem.

[16] Wright DC, Hucker KA, Holloszy JO, Han DH (2004). Ca2+ and AMPK both mediate stimulation of glucose transport by muscle contractions.. Diabetes.

[17] Witczak CA, Jessen N, Warro DM, Toyoda T, Fujii N (2010). CaMKII regulates contraction- but not insulin-induced glucose uptake in mouse skeletal muscle.. Am J Physiol Endocrinol Metab.

[18] Witczak CA, Fujii N, Hirshman MF, Goodyear LJ (2007). Ca2+/calmodulin-dependent protein kinase kinase-alpha regulates skeletal muscle glucose uptake independent of AMP-activated protein kinase and akt activation.. Diabetes.

[19] Mitsumoto Y, Burdett E, Grant A, Klip A (1991). Differential expression of the GLUT1 and GLUT4 glucose transporters during differentiation of L6 muscle cells.. Biochem Biophys Res Commun.

[20] Ostenson CG, Khan A, Abdel-Halim SM, Guenifi A, Suzuki K (1993). Abnormal insulin secretion and glucose metabolism in pancreatic islets from the spontaneously diabetic GK rat.. Diabetologia.

[21] Abdel-Halim SM, Guenifi A, Luthman H, Grill V, Efendic S Impact of diabetic inheritance on glucose tolerance and insulin secretion in spontaneously diabetic GK-wistar rats.. Diabetes.

[22] Tanishita T, Shimizu Y, Minokoshi Y, Shimazu T (1997). The beta3-adrenergic agonist BRL37344 increases glucose transport into L6 myocytes through a mechanism different from that of insulin.. J Biochem.

[23] Lindquist JM, Fredriksson JM, Rehnmark S, Cannon B, Nedergaard J (2000). Beta 3- and alpha1-adrenergic Erk1/2 activation is src- but not gi-mediated in brown adipocytes.. J Biol Chem.

[24] Hutchinson DS, Bengtsson T (2006). AMP-activated protein kinase activation by adrenoceptors in L6 skeletal muscle cells: Mediation by alpha1-adrenoceptors causing glucose uptake.. Diabetes.

[25] Shabalina IG, Hoeks J, Kramarova TV, Schrauwen P, Cannon B (2010). Cold tolerance of UCP1-ablated mice: A skeletal muscle mitochondria switch toward lipid oxidation with marked UCP3 up-regulation not associated with increased basal, fatty acid- or ROS-induced uncoupling or enhanced GDP effects.. Biochim Biophys Acta.

[26] Grynkiewicz G, Poenie M, Tsien RY (1985). A new generation of Ca2+ indicators with greatly improved fluorescence properties.. J Biol Chem.

[27] Hutchinson DS, Csikasz RI, Yamamoto DL (2007). Diphenylene iodonium stimulates glucose uptake in skeletal muscle cells through mitochondrial complex I inhibition and activation of AMP-activated protein kinase.. Cell Signal.

[28] Duchen MR (2000). Mitochondria and calcium: From cell signalling to cell death.. J Physiol.

[29] Niu W, Bilan PJ, Ishikura S, Schertzer JD, Contreras-Ferrat A (2010). Contraction-related stimuli regulate GLUT4 traffic in C2C12-GLUT4myc skeletal muscle cells.. Am J Physiol Endocrinol Metab.

[30] Lanner JT, Bruton JD, Katz A, Westerblad H (2008). Ca(2+) and insulin-mediated glucose uptake.. Curr Opin Pharmacol.

[31] Bruton JD, Katz A, Westerblad H (1999). Insulin increases near-membrane but not global Ca2+ in isolated skeletal muscle.. Proc Natl Acad Sci U S A.

[32] Chen X, Yang L, Oppenheim JJ, Howard MZ (2002). Cellular pharmacology studies of shikonin derivatives.. Phytother Res.

[33] Shoelson SE, Herrero L, Naaz A (2007). Obesity, inflammation, and insulin resistance.. Gastroenterology.

